# Architectural Distortion-Based Digital Mammograms Classification Using Depth Wise Convolutional Neural Network

**DOI:** 10.3390/biology11010015

**Published:** 2021-12-23

**Authors:** Khalil ur Rehman, Jianqiang Li, Yan Pei, Anaa Yasin, Saqib Ali, Yousaf Saeed

**Affiliations:** 1 The School of Software Engineering, Beijing University of Technology, Beijing 100024, China; rehmankhalilur@emails.bjut.edu.cn (K.u.R.); lijianqiang@bjut.edu.cn (J.L.); yasinanaa@emails.bjut.edu.cn (A.Y.); alisaqib@emails.bjut.edu.cn (S.A.); b20196112w@emails.bjut.edu.cn (Y.S.); 2 Beijing Engineering Research Center for IoT Software and Systems, Beijing 100124, China; 3 Computer Science Division, University of Aizu, Aizuwakamatsu 965-8580, Fukushima, Japan

**Keywords:** architectural distortion, image processing, depth-wise convolutional neural network, breast cancer, mammography

## Abstract

**Simple Summary:**

Breast cancer is leading cancer increases the death rate in women. Early diagnosis of breast cancer in women can save their lives. The current study proposed a novel scheme to detect architectural distortion from mammogram images to predict breast cancer using a deep learning approach. Results are evaluated on a public and a private dataset which may help to improve the diagnostic ability of breast cancer of radiologists and doctors in daily clinical routines. Furthermore, the proposed method achieved maximum accuracy as compared with previous approaches. This study can be interesting and valuable in the healthcare predictive modeling domain and will add a real contribution to society.

**Abstract:**

Architectural distortion is the third most suspicious appearance on a mammogram representing abnormal regions. Architectural distortion (AD) detection from mammograms is challenging due to its subtle and varying asymmetry on breast mass and small size. Automatic detection of abnormal ADs regions in mammograms using computer algorithms at initial stages could help radiologists and doctors. The architectural distortion star shapes ROIs detection, noise removal, and object location, affecting the classification performance, reducing accuracy. The computer vision-based technique automatically removes the noise and detects the location of objects from varying patterns. The current study investigated the gap to detect architectural distortion ROIs (region of interest) from mammograms using computer vision techniques. Proposed an automated computer-aided diagnostic system based on architectural distortion using computer vision and deep learning to predict breast cancer from digital mammograms. The proposed mammogram classification framework pertains to four steps such as image preprocessing, augmentation and image pixel-wise segmentation. Architectural distortion ROI’s detection, training deep learning, and machine learning networks to classify AD’s ROIs into malignant and benign classes. The proposed method has been evaluated on three databases, the PINUM, the CBIS-DDSM, and the DDSM mammogram images, using computer vision and depth-wise 2D V-net 64 convolutional neural networks and achieved 0.95, 0.97, and 0.98 accuracies, respectively. Experimental results reveal that our proposed method outperforms as compared with the ShuffelNet, MobileNet, SVM, K-NN, RF, and previous studies.

## 1. Introduction

Breast cancer is leading cancer worldwide in 2020, with 11.7% overall reported cases per world health organization [1] and one of the major causes of death in women. The mortality rate was increased from 6.6% to 6.9% this year due to breast cancer. Initially, these breast cancer tumors are screened on an X-ray machine for breast cancer diagnosis and manually interpreted by the radiologist to predict benign and malignant tumors. Screening methods such as ultrasound, and mammography are used to diagnose breast cancer, while the standard screening method is mammography at the early stage. Computer-aided diagnostic systems automatically detected abnormal regions in mammograms to help radiologists and doctors detect disease in less time to avoid unnecessary biopsies [2].

Breast composition containing attenuating tissue is an essential element for evaluating mammogram reports to predict malignant and benign cases. Architectural distortion (AD) is the third most suspicious appearance on a mammogram representing abnormal regions that can be found visible on mammography projection [3]. The main parameters such as global asymmetry, focal asymmetry, and developing asymmetry of tissue can be calculated using machine and deep learning algorithms to track AD in mammograms. Asymmetries are the isodense tissues obscured by adjacent fibro glandular mass, representing true malignancy in mammograms. Architectural distortion tracking from mammograms is very difficult due to its subtle and varying asymmetry on breast mass and small size. Therefore, the manual interpretation of architectural distortion is a challenging task for radiologists to figure out abnormalities during the examination of mammograms. The leading types of cancer that can present architectural distortion on mammography are invasive lobular carcinoma (ILC) and invasive ductal carcinoma (IDC). The ILC and IDC on mammography having a star-shaped pattern are likely to be malignant, while the complex and radial sclerosing lesions architectural distortion having larger than 1 cm is probably benign [4].

Several studies reported hand-crafted feature extraction techniques on mammogram images for AD ROI classification using machine learning and deep learning [5]. These methods successfully achieved remarkable accuracy in the diagnosis of breast cancer. However, many factors are involved in detecting architectural distortion, such as tinny size, subtle appearance inside mass, shape, noise, imaging artefact from digital mammograms. Due to a limited number of studies that reported AD ROI’s classification in the literature, this primarily discusses the most relevant studies in the first phase. The second phase discusses deep learning, machine learning, and mass segmentation, to determine the limitations of predicting breast cancer. There are many limitations in these studies for detecting architectural distortion ROIs and classification. For example, Murali S. et al. [6] proposed a model-based approach to detect architectural distortion from mammograms and classify with a support vector machine to achieve 89.6 accuracy. A total of 150 ROI’s were selected from the DDSM dataset to evaluate the performance. Banik et al. [7] employed the gobar filter and phase portrait analysis method to detect architectural distortion in prior mammograms by evaluating 4224 ROI’s from a private dataset and achieved 90% sensitivity at 5.7 FP/image. J. et al. [8] presented a two-step method such as detecting ROIs with potential AD on analyzing the Gabor filter and recognizing AD’s using a 2D Fourier transform. Experimental results were evaluated on 33 mammograms containing AD’s from DDSM and obtained 83.50 accuracy. All three authors employed Gabor filter to the texture feature analysis of images while locating the boundary of ADs ROIs was still a limitation. As a result, these hand-crafted feature extraction methods decrease the computational time and affect the model’s classification accuracy.

The classification of AD ROIs based on texture analysis model using support vector machine was implemented on mammogram images by Kamra A. et al. [9]. The texture analysis ROIs were selected from the digital database for screening mammography (DDSM) dataset to evaluate the model’s performance and reported 92.94% accuracy. Liu et al. [10] employed a new method for architectural distortion ROIs recognition based on texture features from gray-level co-occurrence matrix (GLCM) matrix, spiculated and entropy features from mammogram images, and the sparse representation classifier was used for the classification of ROIs. The performance of the model was evaluated on the DDSM dataset by obtaining 91.79 accuracy. Ioana B. et al. [11] proposed radiomic analysis of contrast-enhanced spectral mammography approach for breast cancer prediction and classification using k-nearest neighbors (K-NN). Another radiomic feature reduction approach was proposed by Raffaella M. et al. [12] for mammogram classification to predict breast cancer. D. H. et al. [13] proposed a micro-pattern texture descriptor for the detection of architectural distortion from mammogram images using a local binary pattern, local map pattern, and haralick’s descriptors. A total of 400 ROIs from the full-field digital mammography (FFDM) dataset were selected for the evaluation of the model and achieved 83% accuracy. Casti P. et al. [14] was introduced a new paradigm to detect AD track in digital breast tomosynthesis (DBT) exam by using a cross-cutting approach exploiting 3D imaging modality. The proposed approach achieves 0.9 sensitivity after evaluating the model on 37 sets of DBT from the FFDM dataset. Palma et al. [15] presented a fuzzy contrary-based approach for detecting masses and architectural distortion from digital breast tomosynthesis.

Another essential factor is noise removal from ADs ROIs which was still a limitation with these traditional methods. Moreover, all of the studies were employed traditional machine learning algorithms, which were limited to the lower classification accuracy. The architectural distortion star shapes heterogeneous pattern detection inside the denser mass using the texture analysis was still a limitation. Cai et al. [16] employed a method for identifying architectural distortion in mammogram images using a dense net deep neural network to train the image net model for breast mass dataset to classify the breast masses. Bahl et al. [17] was presented a retrospective review for the presence of architectural distortion on mammogram images and concluded that the presence of architectural diction on mammography has the chance of malignancy in approximately three fourth of the cases. Shu et al. [18] proposed a region-based pooling structure using a deep convolutional neural network to classify mammogram images. The whole region of images as an input to a deep neural network is limited to identifying the subtle location of ADs inside denser breast masses. Conventional deep neural networks only use a single channel for image feature maps which is not limited to neural networks but decreases the overall modal accuracy.

The current study investigated the gap to detect architectural distortion ROIs from mammograms using computer vision techniques. This study employed a depth-wise 2D V-net 64 convolutional neural network to classify these architectural distortion ROIs into benign and malignant ADs. With this approach, the above limitation is no longer. Computer vision is a powerful technology for removing the noise and detecting the object from hidden star-shape patterns. The Depth-wise neural network uses each input channel for creating a feature map that increases the modal efficiency and accuracy. Therefore, this study aim to develop a computer-aided diagnostic system using computer vision and a deep learning model to classify architectural distortions ROIs from digital mammograms at early stages.

The principal outcome of our study is reported as follows:

- Proposed an automated computer-aided diagnostic system based on architectural distortion using computer vision and depth-wise deep learning techniques to predict breast cancer from digital mammograms. Applied the image pixel-wise segmentation using a computer vision algorithm to extract architectural distortion ROIs from the digital mammogram image in the first phase.
- In the second phase, employed a depth-wise V-Net 64 convolutional neural network to extract automatic features from ADs ROIs and classify them into malignant and benign ROIs. Moreover, use machine learning and deep learning algorithms, such as shuffelnet, mobilenet, support vector machine, k-nearest neighbor, and random forest, to classify these ROIs.
- Proposed method obtained higher accuracy than machine learning and with the previous studies. Furthermore, evaluated proposed model with other metrics to enhance the diagnostic ability of the model.
- Evaluated the proposed method on three datasets, the local private PINUM and publicly available CBIS-DDSM and DDSM dataset that makes a fair comparison of the proposed model with others.

## 2. Related Works and Techniques

### 2.1. Conventional Deep Learning Mammogram Classification

The researchers presented several computer-aided diagnostic systems using deep convolutional neural networks to predict breast cancer from digital mammograms. Studied that reported deep learning algorithms for the classification of mammogram images herein briefly reported. Feature fusion bases-deep CNN was applied using extreme learning machines to predict breast cancer from mammograms by wang et al. [19]. An improved ResNet-based convolutional neural network was employed to the classification of mammogram images and significantly improve the area under the curve by Wu et al. [20]. Khan et al. [21] developed multi-view feature fusion-based CAD to detect abnormal and normal patterns from mammograms using a deep neural network to increase the accuracy in breast classification. On segmentation of the pectoral muscle-based approach using a deep convolutional neural network was developed by Soleiman et al. [22] to classify mammogram images. Hao et al. [23] presented an automated framework for identifying mislabeled data using cross-entropy and metric function, and the model was trained using a deep convolutional neural network to improve the classification performance. Sun et al. [24] was presented with an automated computer-aided diagnostic system based on a multimodal deep neural network for the integration of multi-dimensional data to prognosis prediction of breast cancer.

A region of interest-based approach was employed by Guan et al. [25] using u-net deep convolution neural network for locating asymmetric patterns to the diagnosis of breast cancer in digital mammograms. The generative adversarial neural network employed for tumor segmentation from digital mammogram by Singh et al. [26]. Song R. et al. [27] developed a combined feature-based model using a deep convolutional neural network for the classification of breast masses into normal, benign, and malignant classes. To overcome the drawbacks of pixel-wise segmentation of mammogram images, Shen et el. [28] was presented a hierarchical model using a deep convolutional neural network and fuzzy learning for breast cancer diagnosis. Guan et al. [29] applied a generative adversarial network for ROIs cropping from digital mammograms, and then the deep convolutional neural network was implemented for the classification of normal and abnormal ROIs. An improved dense net deep learning model was proposed by Li et al. [30] to classify benign and malignant mammograms. A whole image classification based-method was built using a deep neural network using by Iones et al. [31]. Falcon et al. [32] was employed transfer learning techniques to predict abnormalities in digital mammograms with a deep mobile net neural network.

Gnana S. et al. [33] developed a computer-aided diagnostic system using a deep convolutional neural network to classify malignant and benign masses. A deep active and self-paced learning-based framework was emphasized for detecting breast mass from digital mammograms by Shen et al. [34] to reduce the annotation effort for radiologists. Shen et al. [35] presented a method for lesion segmentation and disease classification using a mixed-supervision-guided residual u-net deep learning modal. Shayma A.H et al. [36] propose a novel method for cancer detection from breast mass using feature matching of different regions by applying maximally stable extremal regions. A hybrid deep learning-based framework was employed by Wang et al. [37] for the classification of breast mass for multi-view data. Wang et al. [38] employed a multi-level nested pyramid deep neural network to segment breast mass to classify malignant and benign classes using a public dataset. Birhanu et al. [39] proposed a breast density classification method to predict cancer from digital mammograms using a deep convolutional neural network. Rehman et al. Proposed a computer vision based deep learning method for the classification of microcalcification ROIs into malignant and benign classes.

### 2.2. Conventional Machine Learning Mammogram Classification

Machine learning modalities such as SVM, KNN, and random forest were adopted to classify digital mammograms to diagnose breast cancer. Machine learning-based classification CAD systems used hand-crafted feature extraction techniques, which are computationally slow and reduce the performance model. Fan et al. [40] proposed a novel method based on single-nucleotide polymorphism to predict breast cancer risk by extracting architectural distortion features from mammograms. Loizidou et al. [41] presented subtraction of temporally sequential mammogram technique to detect microcalcification clusters and classification performed using a support vector machine. The breast boundary is eliminated with the thresholding technique, and a machine learning-based hybrid model is proposed to classify breast mammograms into malignant and benign classes by Zebari et al. [42]. A computer-aided diagnostic system was built to generate an image feature map using fast Fourier transforms on digital mammograms by Heidar et al. [5]. Chakaraborty et al. [43] presented a machine learning-based hybrid approach for automatic detection of mammographic masses using low-to-high level intensity thresholding and performed classification using FLDA, Bayesian, and ANN. Beham et al. [44] applied wavelet transforms for feature extraction from the digital mammogram, and the K-nearest neighbor algorithm was employed for classification into benign and malignant classes. Liu et al. [45] was proposed a novel approach for breast cancer prediction, which employed information gain simulated annealing genetic algorithm for feature selection and const sensitive support vector machine for classification. Another support vector machine-based approach was employed by Yang et al. [46] to diagnose breast tumors using textual features from mammogram images. Obaidullah et al. [47] presented an image descriptor-based approach for mammogram mass classification using a random forest algorithm. Saqib et al. presented the comparison of machine learning techniques for the prediction of multi-organ cancers.

## 3. Materials

### Databases

**Figure 1:**
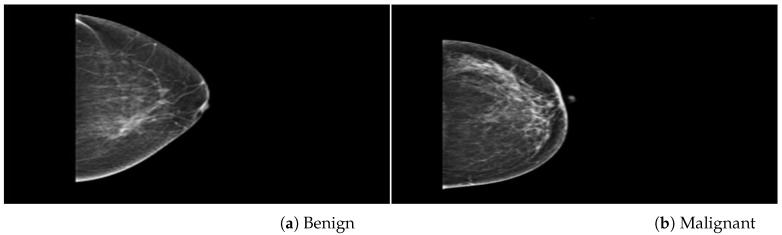
An example of breast mammogram images from PINUM dataset. (**a**) The Benign image (**b**) The Malignant image verified by the Expert radiologist.

This study validated the proposed method on three databases, the PINUM (Punjab institute of nuclear medicine) [48], the CBIS-DDSM (curated breast imaging digital database for screening mammography) [49] and DDSM (digital database for screening mammography) [50]. The PINUM private dataset was collected from a local hospital in Pakistan with the approval of diagnostic imaging nuclear medicine and radiology. A total of 289 patient data in the form of DICOM (Digital Imaging and Communications in Medicine) images were collected ranging age between 32-73 with a mean age of 48.5 years. The dataset includes 577 original images containing 425 benign and 152 malignant images with MLO (mediolateral-oblique) and CC (craniocaudal) views at the resolution of 4096×2047 are shown in Figure 1. The proposed study is based on architectural distortion, so that the validation set of mammogram images is labeled by the radiologist for benign and malignant architectural distortion ROIs. A total of 150 AD ROIs are cropped from full mammograms for validating the training set with the proposed algorithm. The radiologist team consisted of two members, one being a senior radiologist and physicist holding a Ph.D. degree in nuclear medicine with 10 years of experience and the second being a junior radiologist with a Master’s degree in radiology. The mammography exam of the PINUM dataset was acquired with Hologic 2D, 3D mammography. The PINUM dataset images have MLO and CC views. The size of the PINUM dataset was artificially inflated using augmentation techniques up to 3462 images.

**Figure 2:**
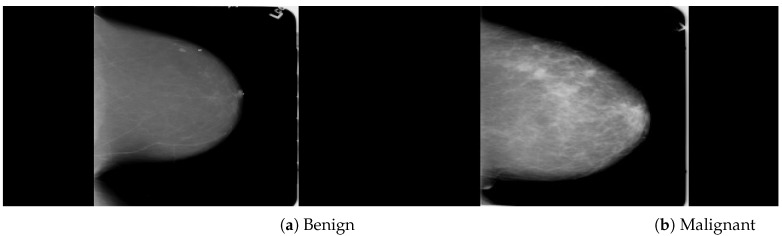
An example of breast mammogram images from CBIS-DDSM dataset. (**a**) The Benign image (**b**) The Malignant image with verified pathology information.

**Figure 3:**
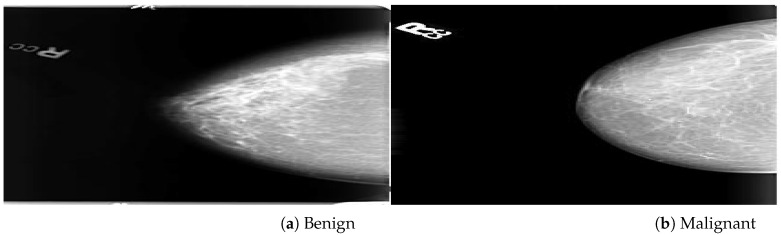
An example of breast mammogram images from DDSM dataset. (**a**) The Benign image (**b**) The Malignant image with verified ground truth information.

The CBIS-DDSM (digital database for screening mammography) was a public dataset and enhanced version of the DDSM dataset provided by the University of Florida. The mammogram images are in DICOM files at the complete mammography and abnormality levels. Both MLO and CC views of the mammograms are included in the full mammography pictures. Abnormalities are represented as binary mask images that are the same size as the mammograms they are connected with. The ROI of each anomaly is defined by these mask images. Within each mammogram’s abnormality mask, users may make an element-by-element selection of pixels. Due to the unavailability of AD ROIs in the CBIS-DDSM dataset, our radiologist team labeled ADs ROIs manually on full mammogram images. A total of 200 AD ROIs are cropped from full mammograms for validation. We included 3568 mammogram images, including 1740 benign and 1828 malignant images with MLO and CC views, as shown in Figure 2. The DDSM is a public dataset provided by Massachusetts General Hospital, Wake Forest University School of Medicine, and Sacred Heart Hospital and maintained by the University of Florida. The DDSM datasets contain 2500 studies including normal, benign, and malignant cases. Each study comprises two images of the breast as well as some patient data such as age at the time of the study, ACR breast density rating, and subtlety rating for abnormalities. Suspicious lesions in images are correlated with pixel-level ground truth information about their positions and kinds. The DDSM datasets contain 200 ADs ROIs of benign and malignant images. In this study, the predefined ADs are considered validation test datasets. A total of 5500 images (2500 benign, 3000 malignant) were included for training and testing the neural networks from the DDSM dataset. Figure 3 shows benign and malignant mammogram images from DDSM dataset. A detailed description of the datasets is in Table 1.

## 4. Methods

### 4.1. Proposed Method

**Figure 4:**
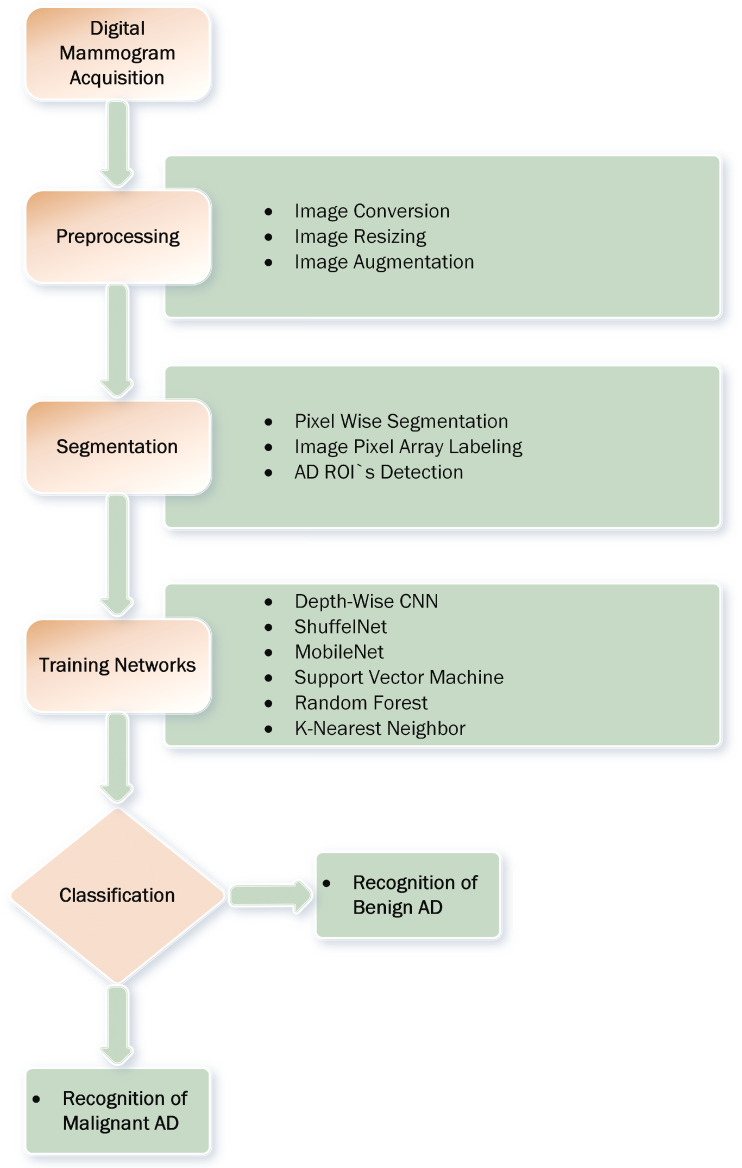
The proposed mammogram classification framework pertains to four steps: image preprocessing and augmentation, pixel wise segmentation and image pixel array labeling, architectural distortion ROI’s detection, training deep learning, and machine learning networks to classify AD’s ROIs into malignant and benign classes.

In this study, proposed a novel approach for the classification of architectural distortion using a depth-wise 2D V-net 64 convolutional neural network. The proposed method pertains to two steps: in the first step, a computer vision algorithm is used for AD ROIs extraction from digital mammogram images. In the second step, the extracted AD ROIs are classified using a depth-wise convolutional neural network. The proposed method can achieve higher accuracy than the deep machine learning methods such as shuffelnet, mobilenet, support vector machine, k-nearest neighbor, and random forest and previous studies. Furthermore, evaluate the performance of the proposed method with other evaluation metrics such as f1_score, precision, recall, sensitivity, specificity, and area under the curve (AUC). The proposed framework of proposed method for mammogram classification based on architectural distortion is presented in Figure 4. The details about the proposed methodology are determined in subsequent sections.

### 4.2. Image Preprocessing

Image conversion and resizing are employed in the preprocessing step to remove noise, artifacts, and irrelevant information. The original mammograms were acquired from three databases such as the PINUM [48] local database and the public database CBIS-DDSM [49], and DDSM [50]. The original databases PINUM and CBIS-DDSM were in the DICOM (digital imaging and communications in medicine) format containing images and patient data. In the first step, the DICOM images are converted into PNG format using an automated OpenCV conversion method, and the patient data is stored in a CSV file. The image preprocessing Algorithm 1 is reported below the complete steps. The converted PNG breast mammogram images are very high-resolution images with a 4096×2047 width and height. We employed the automatic image resizing method with a two-integer argument width and height by downsizing resolution up to 320×240 pixels to make fixed-size images before training a deep convolutional neural network. The DDSM database images are in gif format and converted into PNG format using the automated conversion method.
**Algorithm 1** Image preprocessing algorithm 1.Step 1: Select the DICOM file using read method.;Step 2: Read DICOM Description values.;Step 3: Create input vector of DICOM file;Step 4: Write image description;Step 5: Read patient data;Step 6: Read image pixel values;Step 7: Apply image function zoom in/out;Step 8: Apply Linear Interpolation function;Step 9: Create new input vector for new format;Step 10: Replace Pixels DICOM format to PNG;Step 11: Write patient data;Step 12: Save converted image and patient data;Step 13: Display PNG image;

### 4.3. Image Augmentation

**Figure 5:**
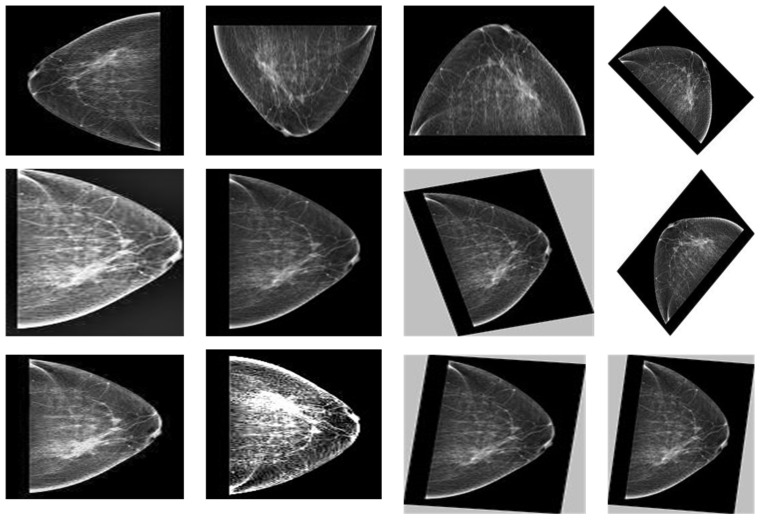
The augmented images of PINUM dataset starting from original to augmented images.

Deep learning is a data-driven method so that the small size of data and non- standardization are the main challenges for the generalization of the model. However, to handle the generalization, overfitting, and improving the robustness of the deep learning model, we artificially inflate the PINUM database five times from the original images to increase the dataset size. The data augmentation techniques such as rotating, flipping, sharpening, d-skew, brightness, and contrast are employed to increase the dataset’s size, as shown in Table 2. In addition, the overfitting and generalization of the deep learning model can be improved by applying augmentation [51]. The mammogram images are rotated at 45, 90, 135, 180, and 360 degrees and return a new object of the rotated images within a described resolution to increase dataset size up to 3462. Moreover, we rotated a single mammogram at five angles that produce five rotated images and one original image and employed augmentation methods, as shown in Figure 5. The volume of the CBIS-DDSM and the DDSM dataset is 3568, 5500 images; therefore, the data augmentation was not employed on both datasets as the modal overfitting and generalization was not a challenging issue.

### 4.4. Pixel Wise Segmentation

The image pixel-wise segmentation method maps each pixel of the image that belongs to the image’s object or shape and gives a label. M. Wang et al. [52] employed image path-based pixel segmentation using a label fusion algorithm. The image pixel-wise segmentation method maps each pixel of the image that belongs to the image’s object or shape and gives a label. Pixels have the same attribute locating an object of the image. Computer vision is a powerful technology for detecting objects as compared with other object detection techniques. Employed a computer vision-based object detection technique and create an image pixel array. Each pixel array has labeled with a class label0 and label1. The detailed process is as follow:

1. The image is to be segmented as a targeted image $P={\left(x,y,N\right)}^{w\times h}$, where *P* representing a pixel array vector having *N* elements that has belongs to the specific category as:
  (1) $$\sum_{p}P\in {\left(x,y\right)}^{w\times h}=L\in \left[0,1\right]$$
2. The pixel x∈(x1,x2,⋯w) and y∈(y1,y2,⋯h) represents the vertical *w* and horizontal *h* pixels, where x1 and y1 are the elements of pixel vector. The dot product has performed as:
  (2) P(x,y)=P(x,y).L
3. L∈[0,1] represents each object in a pixel array belonging to classes 0 and 1. The pixel-wise prediction can be improved on which we can generate the segmentation results.

### 4.5. Architectural Distortion ROI’s Detection

**Figure 6:**
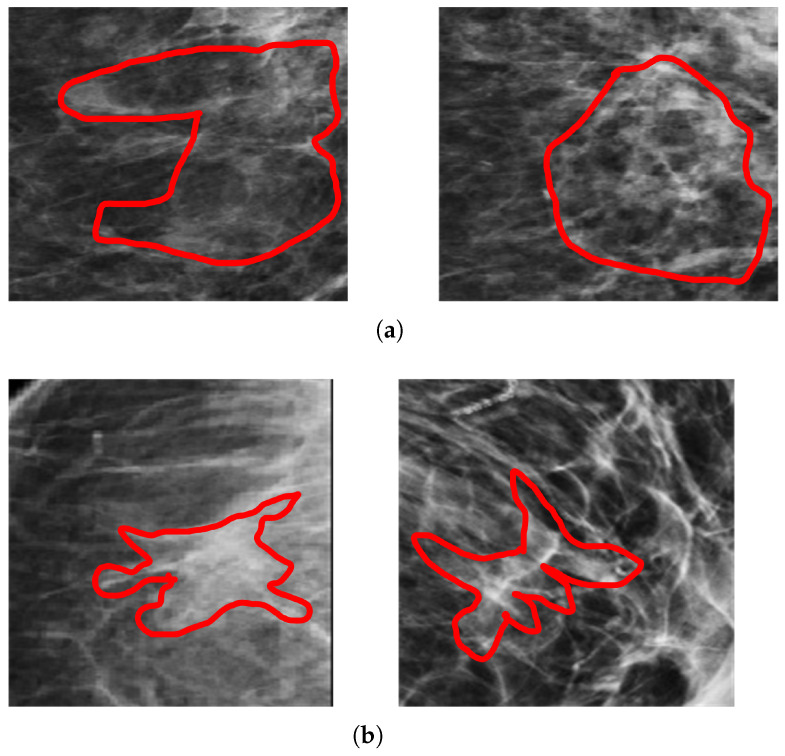
An example of the architectural distortion ROI’s from PINUM dataset by the experts team of radiologists. (**a**) Radial shape (**b**) Star shape.

**Figure 7:**
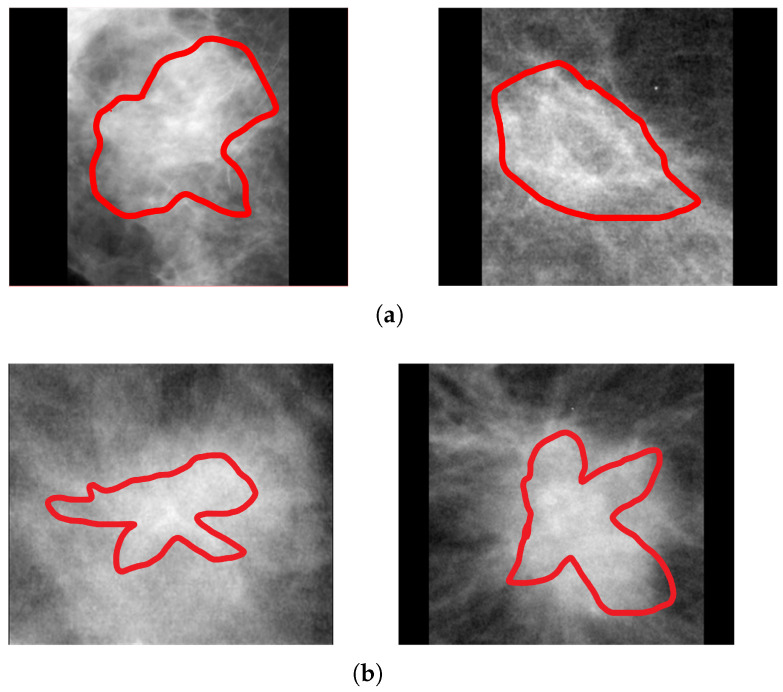
An example of the architectural distortion ROI’s segmentation of CBIS-DDSM dataset by the radiologists. (**a**) Radial shape (**b**) Star shape.

**Figure 8:**
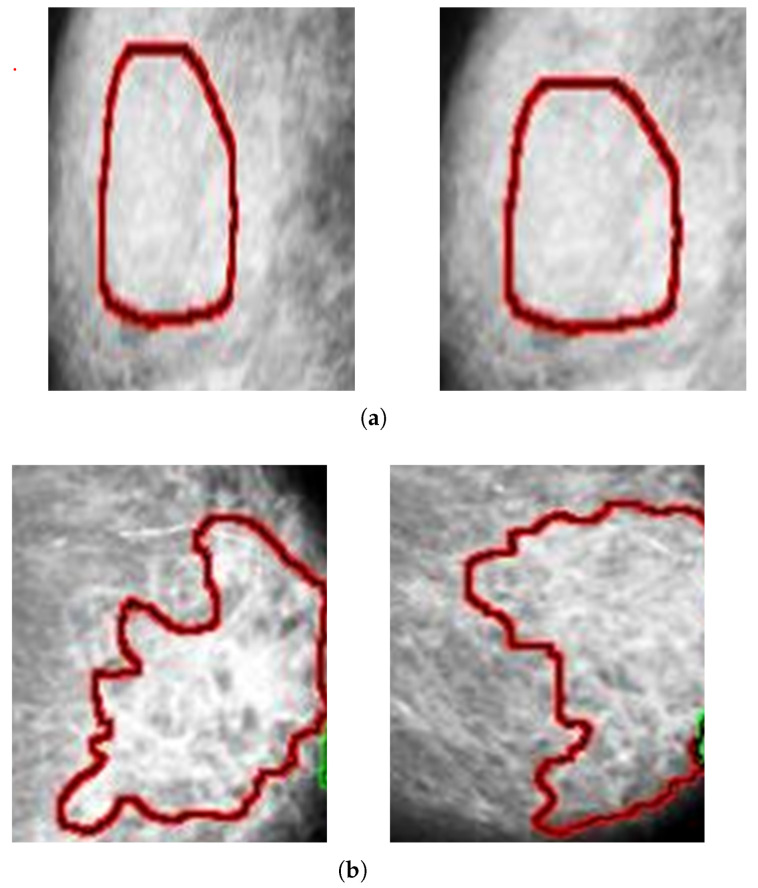
An example of the architectural distortion ROI’s segmentation of DDSM dataset. (**a**) Radial shape (**b**) Star shape.

Architectural distortion is the third most suspicious appearance on a mammogram that represents abnormal regions. Architectural distortion tracking from mammograms is challenging due to its subtle and varying asymmetry on breast mass and small size. The architectural distortion associated with ILC or IDC on mammography represents the abnormality, and having a star-shaped pattern is likely to be malignant, while the complex and radial sclerosing lesions architectural distortion having larger than 1 cm is probably benign [4]. Employed computer vision-based pixel-wise segmentation for the detection of AD ROIs from digital mammograms. In the first step, the computer vision object detection algorithm was applied to create a segmented pixel array. In the second step, the area having a star shape pattern and larger radios than 1 cm was considered as ADs ROIs. The segmented architectural distortion ROIs input to the dept-wise convolutional neural network for classification. Figure 6, Figure 7 and Figure 8 presented segmented benign and malignant ROIs from the PINUM, CBIS-DDSM datasets and DDSM. Moreover, we pertain to the same procedure for the segmentation of AD ROIs from the CBIS-DDSM dataset. The automated segmented ROIs are validated with manually marked ADs ROIs by the radiologist team. The DDSM dataset has predefined ground truth ADs ROIs and is included in the validation dataset. Samreen et al. [53] presented an imaging evaluation management algorithm on architectural distortion detection from digital breast tomosynthesis.

### 4.6. Depth-Wise-CNN Architecture

**Figure 9:**
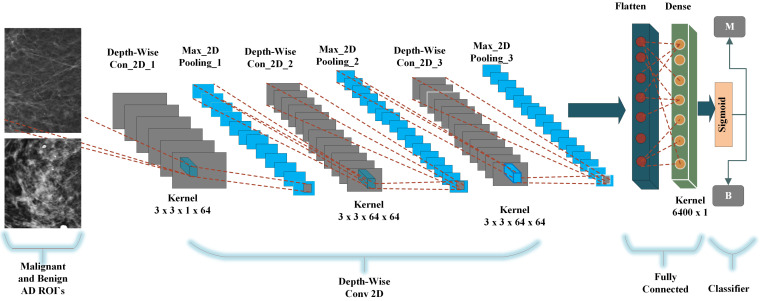
The proposed depth-wise CNN architecture for the classification of benign and malignant architectural distortion ROIs.

A deep convolutional neural network using a computer vision-based method has improved pattern recognition and architectural distortion classification. The standard convolutional neural network uses input and output with only width and height parameters. For input with only width and height, the neural network increases the parameters and can be overfitting. Employed a depth-wise 2D convolutional neural network using V-net 64 architecture with three convolutional layers, three max-pooling layers, one fully connected flatten layer, and one dense layer followed by the sigmoid classifier. The depth-wise convolution only uses one input channel for each depth level of input and then performs convolution. The depth-wise convolutional neural network architecture is presented in Figure 9. In the convolutional layer, use a 3×3 kernel using the Relu activation function and the input vector mapping the features to the convolutional layer as $dim\left(image\right)=\left(n_{h},n_{w},n_{c}\right)$ Where $n_{h}$ is the size of height, $n_{w}$ size of width and $n_{c}$ is the number of channels. The input image of the $l^{th}$ layer we use $a^{\left[l-1\right]}$ filters with the size of $\left(n_{h}^{\left[l-1\right]},n_{w}^{\left[l-1\right]},n_{c}^{\left[l-1\right]}\right),a^{\left[0\right]}$. The stride parameter is: $s^{\left[l\right]}$ and the number of filters denoted as $n_{c}^{\left[l\right]}$ where for each $K^{n}$ is size of $\left(f^{\left[l\right]},f^{\left[l\right]},n_{c}^{\left[l-1\right]}\right)$. The activation function ReLu is: $\varphi^{\left[l\right]}$ and the output image is $a^{\left[l\right]}$ with the size of $(n_{h}^{\left[l\right]},n_{w}^{\left[l\right]},n_{c)}^{\left[l\right]}$. Equations (3) and (4) shows the input and output of convolutional layer. For all *n* belongs to $\left[1,2,\ldots ,n_{c}^{\left[l\right]}\right]$.

(3) $$\begin{array}{c} Conv{\left(a^{\left[l-1\right]},K^{n}\right)}_{x,y}=\varphi^{\left[l\right]}(\sum_{i=1}^{n_{h}^{\left[l-1\right]}}\sum_{j=1}^{n_{w}^{\left[l-1\right]}}\sum_{k=1}^{n_{c}^{\left[l-1\right]}}\\ K_{i,j,k}^{n}a_{x+i-1,y+j-1,k}^{l-1}+b_{n}^{l})\\ dim\left(conv\left(a^{\left[l-1\right]},K^{n}\right)\right)=\left(n_{h}^{\left[l\right]},n_{w}^{\left[l\right]}\right) \end{array}$$

(4) $$\begin{array}{c} {}[\varphi^{\left[l\right]}\left(Conv\left(a^{\left[l-1\right]},K^{1}\right)\right),\varphi^{\left[l\right]}\left(Conv\left(a^{\left[l-1\right]},K^{2}\right)\right),\ldots \\ \varphi^{\left[l\right]}\left(Conv\left(a^{\left[l-1\right]},K^{\left(n_{c}^{\left[l\right]}\right)}\right)\right)\\ dim(a^{\left[l\right]}=\left(n_{h}^{\left[l\right]},n_{w}^{\left[l\right]},n_{c}^{\left[l\right]}\right)\\ n_{c}^{\left[l\right]}=numberoffilters \end{array}$$

where *f* is activation, x and y the actual pixels location on height and width dimension of input image. The learning parameters of convolutional layer at $l^{th}$ layers are $\left(f^{\left[l\right]}\times f^{\left[l\right]}\times f_{c}^{\left[l-1\right]}\right)\times n_{c}^{\left[l\right]}$ filters. In the max-pooling layer, uses a 2×2 kernel size to down-sampling the features and the input size is $a^{\left[l-1\right]}$ with the size of $\left(n_{h}^{\left[l-1\right]},n_{w}^{\left[l-1\right]},n_{c}^{\left[l-1\right]}\right),a^{\left[0\right]}$. The filter size of pooling layer is denoted as $f^{\left[l\right]}$ and the pooling function $\phi^{\left[l\right]}$. The Equations (5) and (6) performs the pooling function.

(5) $$\begin{array}{c} a_{x,y,z}^{\left[l\right]}=pool{\left(a^{\left[l-1\right]}\right)}_{x,y,z}=\phi^{\left[l\right]}\\ \left({\left(a_{x+i-1,y+j-1,z}^{\left[l-1\right]}\right)}_{\left(i,j\right)\in {\left[1,2,\ldots f^{\left[l\right]}\right]}^{2}}\right)\\ dim\left(a^{\left[l\right]}\right)=\left(n_{h}^{\left[l\right]},n_{w}^{\left[l\right]},n_{c}^{\left[l\right]}\right)\\ n_{c}^{\left[l\right]}=n_{c}^{\left[l-1\right]} \end{array}$$

where (i,j) belongs to $\left[1,2,\ldots ,\phi^{\left[l\right]}\right]$, x,y are the pixels location and *z* is the input channel. The last fully-connected layer a fine number of neurons as input vector considering the $j^{th}$ nodes of the $i^{th}$ layer can be calculated with Equation (6).

(6) $$\begin{array}{c} Z_{j}^{\left[j\right]}=\sum_{l=1}^{n_{i-1}}w_{j,l}^{\left[i\right]}a_{l}^{\left[i-1\right]}+b_{j}^{\left[i\right]}\\ \to a_{j}^{\left[i\right]}=\varphi^{\left[i\right]}\left(z_{j}^{\left[i\right]}\right) \end{array}$$

The input $a^{\left[i-1\right]}$ the result of the convolutional and pooling layer with the dimensions $\left(n_{h}^{\left[i-1\right]},n_{w}^{\left[i-1\right]},n_{c}^{\left[i-1\right]}\right)$. Finally the 1D flatten layer has the dimensions $\left(n_{h}^{\left[i-1\right]}\times n_{w}^{\left[i-1\right]}\times n_{c}^{\left[i-1\right]},1\right)$. and the nodes are:

$$n_{i-1}=n_{h}^{\left[i-1\right]}\times n_{w}^{\left[i-1\right]}\times n_{c}^{\left[i-1\right]}$$

where $w_{j,l}$ are weights with learned parameters $n_{\left[l-1\right]}\times n_{l}$ parameters at $l^{t}h$ layer. The proposed depth-wise convolutional neural network significantly outperformed without overfitting and achieved the highest accuracy.

### 4.7. Depth-Wise-V-Net64 Training

**Figure 10:**
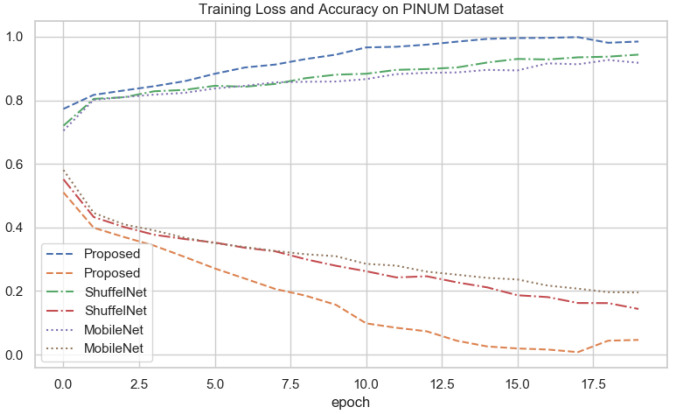
All Deep Networks Training Loss and Accuracy on PINUM Dataset.

**Figure 11:**
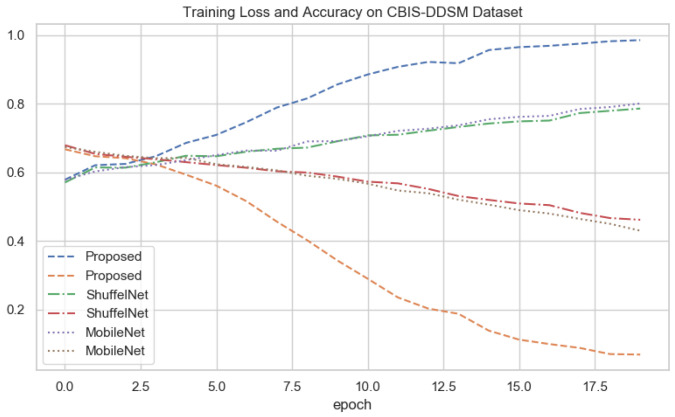
All Deep Networks Training Loss and Accuracy on CBIS-DDSM Dataset.

**Figure 12:**
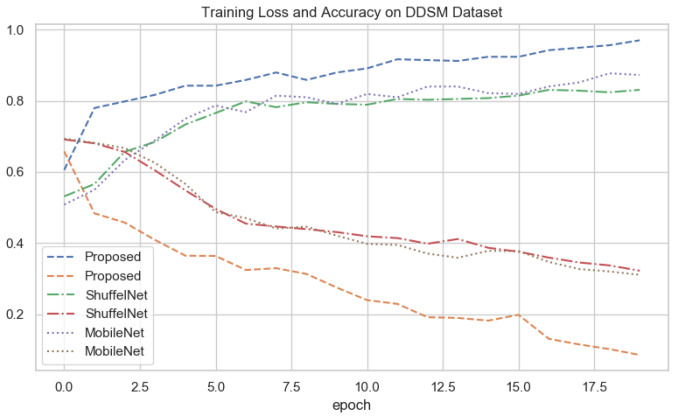
All Deep Networks Training Loss and Accuracy on DDSM Dataset.

The depth-wise 2D convolutional neural network is evaluated on three databases, the local PINUM, the public CBIS-DDSM, and the DDSM dataset. Split the data into the training, testing, and validation data for the proposed deep neural modal. The dataset was randomly divided into 60% for training, 20% for testing, and 20% for cross-validation. For the deep learning model’s regularization and adequate robustness, the data augmentation object is used in our deep learning network for both datasets. Build a depth-wise 2D V-net 64 architecture with three convolutions, three max-pooling, and two fully connected layers for the training of our dataset. The sigmoid classifier has pertained to the classification of malignant and benign ADs ROIs. The epochs size was set 20 to reduce the learning rate by 0.1 factor after every 2.5 epochs, the batch size was 16, and the class weight and "binary_crossentropyloss" function were used to deal with training data imbalance. The proposed deep learning models learning ability was increased as the training ephods increases. Figure 10, Figure 11 and Figure 12 shows that the noise around the data is higher at first layer of network. As well as the modal learns more the noise around the data decreases till the last layer. The training loss continues decreases after the 10th epochs and training accuracy increases and reached up to 100. The training graphs shows that modals learning ability is better and well regularized. The network structure considered in experiments is summarized in Table 3.

### 4.8. Standard Classifiers

ShuffleNet, developed by Magvi Inc, is a highly efficient convolutional neural network architecture optimized for mobile devices with low processing capacity. The new design makes use of two procedures to decrease computing costs while maintaining or improving accuracy and perform groups convolutions pointwise and the Channel Shuffle. The Channel Shuffle is a novel procedure performed to create additional feature map channels, which aids in the encoding of more information and improves the robustness of feature recognition. Group Convolution, introduced in AlexNet, is a form of convolution in which the channels are divided into groups and then the kernel is convolved individually on each group and then re concatenated. This procedure contributes to the retention of existing connections and reduces the connection count.

MonileNet is a deep convolutional neural network that uses a depth-wise separable convolutional neural network. Compared to a network with normal convolutions of the same depth in the nets, it substantially reduces the number of parameters. MobileNet is an open-source neural network provided by Google. The actual difference between the MobileNet design and a conventional CNN is that instead of a single 3×3 convolutional layer followed by the batch norm and ReLU, the MobileNet architecture uses several 3×3 convolutional layers. The mobile nets divide the convolution into a 3×3 depth-wise convolution and a 1×1 point-wise convolution.

Loi et al. [41] presented subtraction of temporally sequential mammogram technique to predict breast cancer using a support vector machine algorithm. To validate the proposed method, perform a classification task using a support vector machine algorithm. A computer vision-based object detection method was employed for architectural distortion ROIs detection in the preprocessing phase. we extracted pixel-wise features using a computer-vision algorithm for creating input to SVM and for other machine learning algorithms. We use the non-linear kernel function in the support vector machine algorithm to classify ADs ROIs. It has been observed that the support vector machine algorithm provides more general results where the number of samples is relatively low [54]. In our SVM model, we employed a 5-fold cross-validation function for the validation of SVM.

K-NN is a supervised machine learning algorithm for binary class, multiclass, and regression problems. Beham et al. [44] applied wavelet transforms for feature extraction from the digital mammogram, and the K-nearest neighbor algorithm was employed for classification into benign and malignant classes. We employed K-NN for binary classification to evaluate and compare the performance of our deep neural network. The image segmentation and ROIs detection method were the same as we use for the SVM algorithm. We set the maximum value for K as 40 and the optimal error rate is 0.17 which shows the K-NN classifier was not overfitted.

Random forest is a supervised machine learning algorithm that ensembles a tree. Obaidullah et al. [47] presented an image descriptor-based approach for mammogram mass classification using a random forest algorithm. In each node of a tree gets a vote for predicting the output. We use a computer vision-based feature selection method for a random forest classifier. We trained a multiple-time random forest classifier to classify ADs’ ROIs, compare it with our proposed method, and observe that random forest performance was low.

### 4.9. Evaluation Metrics

The proposed method was able to classify detected architectural distortion ROIs into malignant and benign classes and significantly improve model accuracy. The performance of the proposed method is evaluated on the local PINUM, the public CBIS-DDSM, and the DDSM database. The evaluation metrics such as accuracy, sensitivity, f1-score, precision, recall, and area under the curve (AUC) are used to assess the performance of the proposed method. The following equations are employed to calculate the accuracy, sensitivity, f1-score, and area under the curve. Accuracy measures the corrected classified sample of the binary class. Sensitivity measures the corrected true-positive cases from false-positive. The area under the curve calculates the ratio between true-positive and false-positive. F1-score can be calculated to compute precision and recall.

(7) $$Accuracy=\frac{TP+TN}{FP+FN+TP+TN}$$

(8) $$Sensitivity=\frac{TP}{TP+FN}$$

(9) $$F1-Score=2*\frac{\left(\frac{TP}{TP+FP}\right)*\left(\frac{TP}{TP+FN}\right)}{\left(\frac{TP}{TP+FP}\right)+\left(\frac{TP}{TP+FN}\right)}$$

(10) $$AUC=\frac{1}{2}*\left(\frac{TP}{TP+FN}+\frac{TN}{TN+FP}\right)$$

where TP: true positive, TN: True negative, FP: False positive, FN: False Negative.

## 5. Results Analysis

The proposed method was designed on scientific fundamentals to predict breast cancer from digital mammograms. The computer vision-based image preprocessing method has pertained to detecting the architectural distortion ROIs from digital mammograms for all models. The experiments were carried out on six pre-trained models (Proposed-CNN, ShuffelNEt, MobileNet, SVM, K-NN, RF) to evaluate the two databases. The experimental results reveal that our proposed method outperforms as compared with other and previous studies.

### 5.1. Experimental Configuration

In the current study, experimental work was performed on google collab GPU, 12 GB RAM, and Windows 10 operating system. All experimental algorithms are implemented in python 3.6 using TensorFlow/Keras library. The computation time was 30 min for training and testing on PINUM datasets, 40 min on the CBIS-DDSM dataset, and 50 min on the DDSM for all neural networks. Furthermore, image preprocessing and augmentation are performed in Python. Pertained to the best hyperparameters, such as batch size, loss function, learning rate, target size, and optimization function, as presented in Table 4.

### 5.2. Comparison between Proposed Method, ShuffelNet, MobileNet and SVM, KNN, RF

The results of the proposed method were compared with well-known three machine learning and two deep learning algorithms. It could be observed that in Table 5, Table 6 and Table 7 the performance of the proposed method was much better than the ShuffelNet, MobileNet, SVM, K-NN, and random forest. The performance of experimental results was evaluated using a five-fold cross-validation test on the PINUM, the CBIS-DDSM, and DDSM datasets. The deep learning models training accuracy and training loss for all datasets has shown in Figure 10, Figure 11 and Figure 12. In Figure 10, after the 7th epochs, the training loss continuously decreases while the training accuracy remains constant over the iterations, while the loss and accuracy of shuffelnet and mobilenet are lower which shows our model perfectly fitted on the PINUM dataset. Figure 11 and Figure 12 for the CBIS-DDSM and DDSM datasets after the 10th epoch, the training loss steadily decreases while the training accuracy remains higher until the last epochs as compared to shuffelnet and mobilenet. The training accuracy on all datasets reaches 99% after the 17th epochs, which indicates that our model was regularized and perfectly fitted.

**Figure 13:**
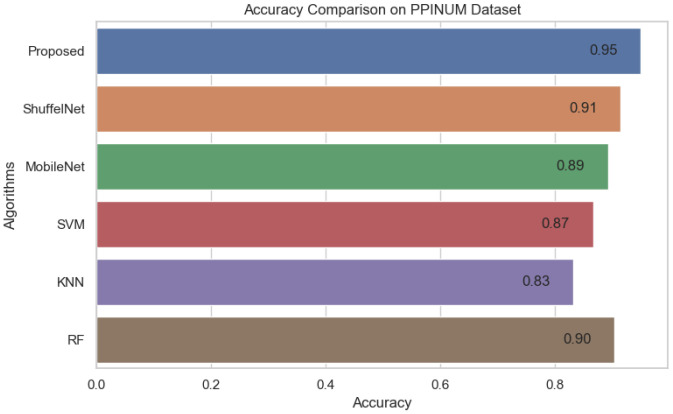
Accuracy Comparison on PINUM Dataset.

**Figure 14:**
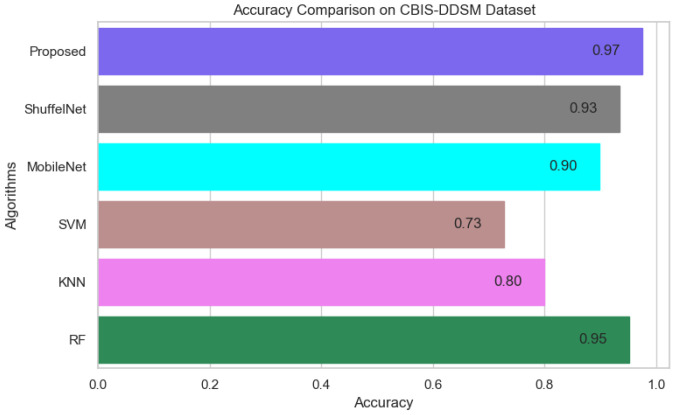
Accuracy Comparison on CBIS-DDSM Dataset.

**Figure 15:**
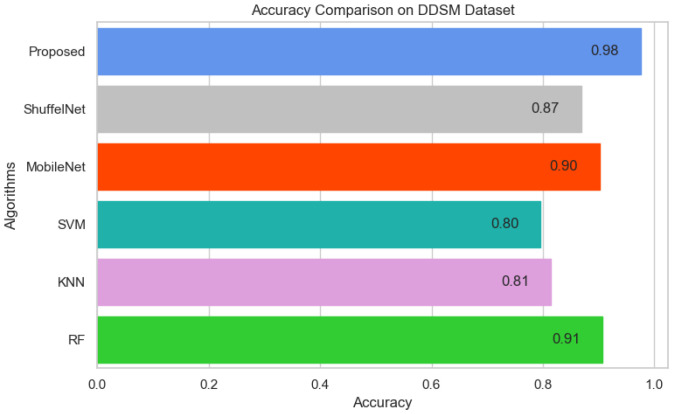
Accuracy Comparison on DDSM Dataset.

Figure 13, Figure 14 and Figure 15 show that the proposed method yielded the best performance and achieved 0.95, 0.97 and 0.98 accuracies on the PINUM, CBIS-DDSM and DDSM datasets, respectively. Shuffelnet, MobileNet, SVM, K-NN, and RF accuracies were 0.91, 0.89, 0.87, 0.83, and 0.90 on the PINUM dataset, 0.93, 0.90, 0.73, 0.80, and 0.95 on the CBIS-DDSM dataset and 0.87, 0.90, 0.80, 0.81 and 0.91 on DDSM dataset. The proposed method achieves 4%, 6%, 8%, 12%, and 5% higher accuracy than ShuffelNet, MobileNet, SVM, K-NN, and RF on the PINUM dataset, 4%, 7%, 24%, 17%, and 2% on the CBIS-DDSM dataset and 11%, 8%, 18%, 17% and 7% on DDSM dataset.

**Figure 16:**
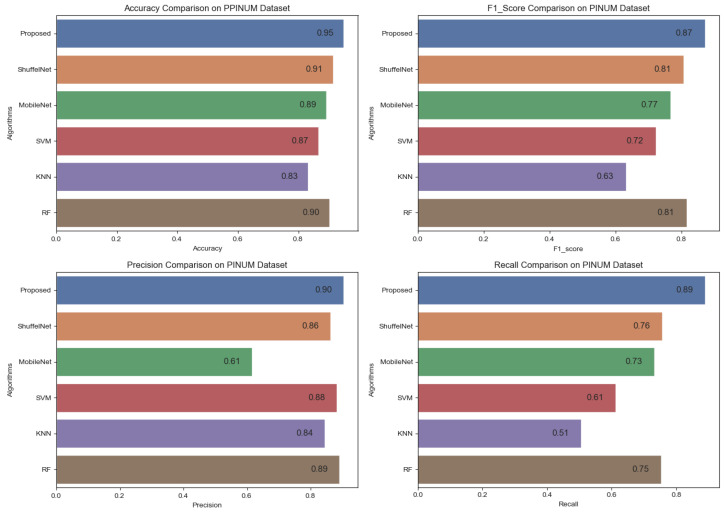
Comparison of Accuracy, F1-Score, Precision and Recall on PINUM Dataset.

**Figure 17:**
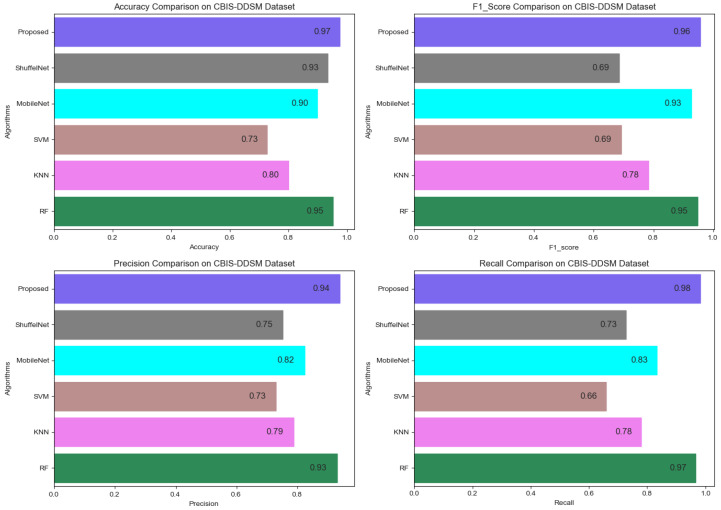
Comparison of Accuracy, F1-Score, Precision and Recall on CBIS-DDSM Dataset.

**Figure 18:**
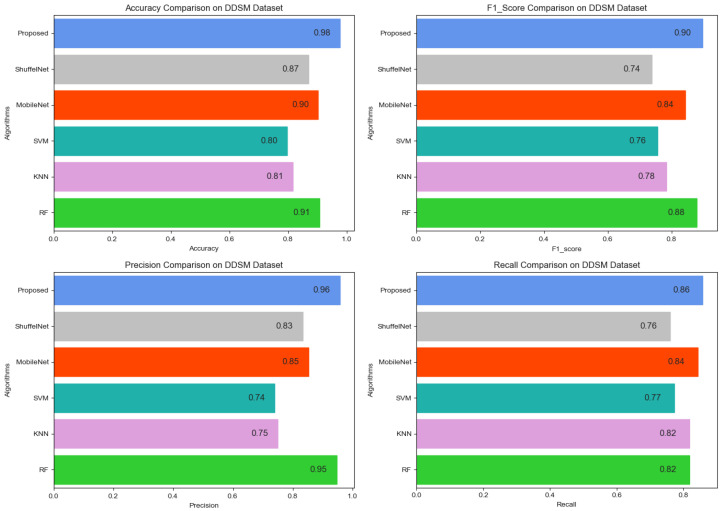
Comparison of Accuracy, F1-Score, Precision and Recall on DDSM Dataset.

Figure 16, Figure 17 and Figure 18 reveals that the proposed method was achieved 0.87, 0.90, 0.89 f1-score, precision, and recall on the PINUM dataset, 0.96, 0.94, and 0.98 on the CBIS-DDSM dataset, and 0.90, 0.96 and 0.86 on DDSM dataset which was higher as compared with ShuffelNet, MobileNEt, SVM, K-NN, and RF, respectively. In addition, the performance of the f1-score of the proposed method was 6%, 10%, 15%, 24%, and 6% higher than ShuffelNet, MobileNet, SVM, K-NN, and RF on the PINUM dataset. Furthermore, f1-score was 27%, 3%, 27%, 18%, and 1% higher than ShuffelNet, MobileNet, SVM, K-NN, and RF on the CBIS-DDSM dataset and 16%, 6%, 14% 12% and 2% on the DDSM dataset. Moreover, the precision and recall of the PINUM dataset of the proposed model was 4%, 29%5, 2%, 6%, 1%, and 13%, 16%, 28%, 38%, 14%, respectively, higher than the ShuffelNet, MobileNet, SVM, K-NN, and random forest. For the CBIS-DDSM and DDSM data set, the proposed method precision and recall performance was 19%, 12%, 21%, 15%, 1% and 25%, 15%, 32%, 20%, 1% and 13%, 11%, 24%, 21%, 1% and 10%, 2%, 9%, 4%, 4% better than the ShuffelNet, MobileNet, SVM, K-NN, and RF.

**Figure 19:**
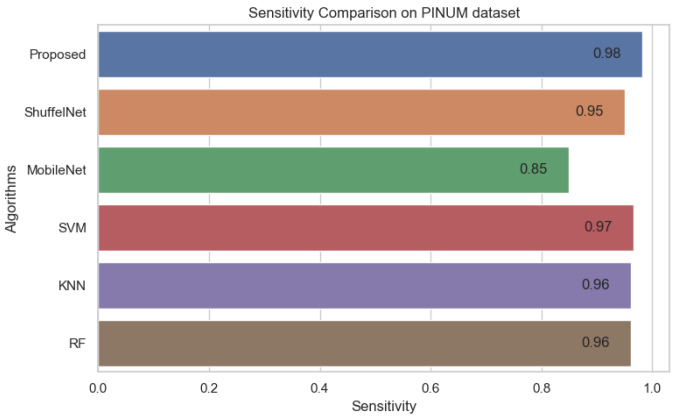
Sensitivity Comparison on PINUM Dataset.

**Figure 20:**
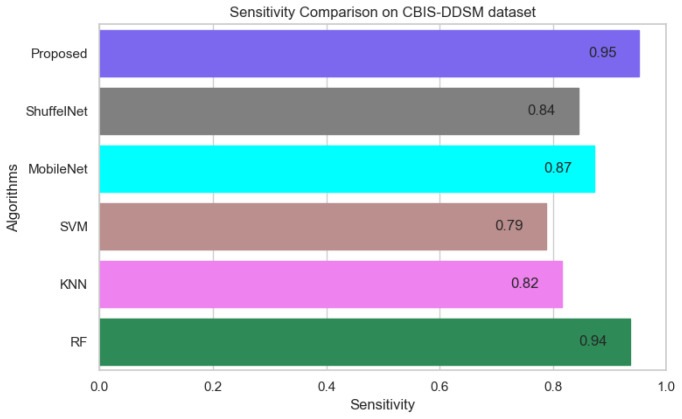
Sensitivity Comparison on CBIS-DDSM Dataset.

**Figure 21:**
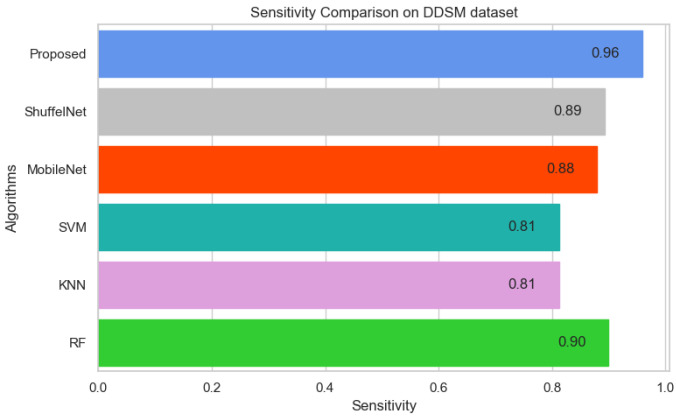
Sensitivity Comparison on DDSM Dataset.

**Figure 22:**
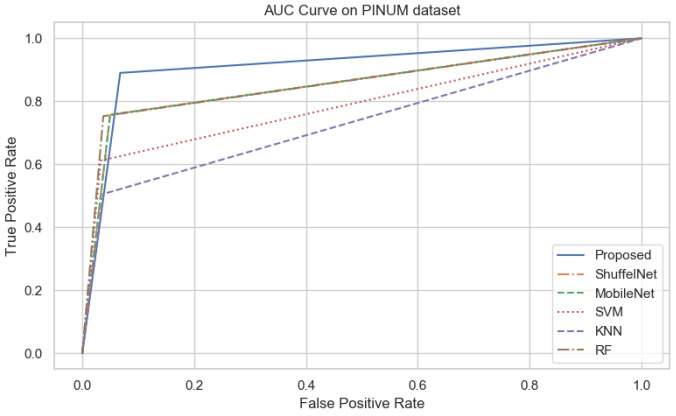
The AUC curves of algorithms on PINUM Dataset.

**Figure 23:**
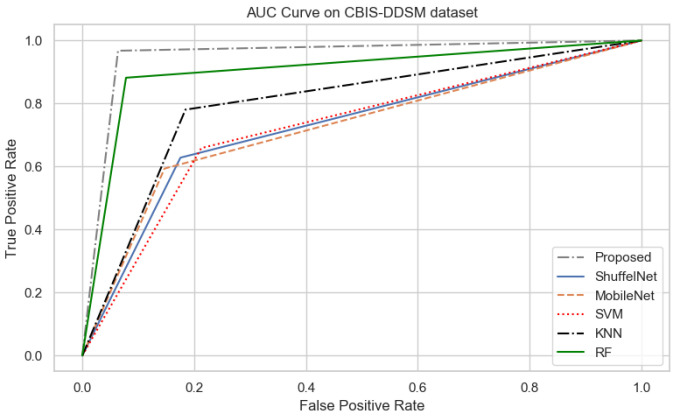
The AUC curves of algorithms on CBIS-DDSM Dataset.

**Figure 24:**
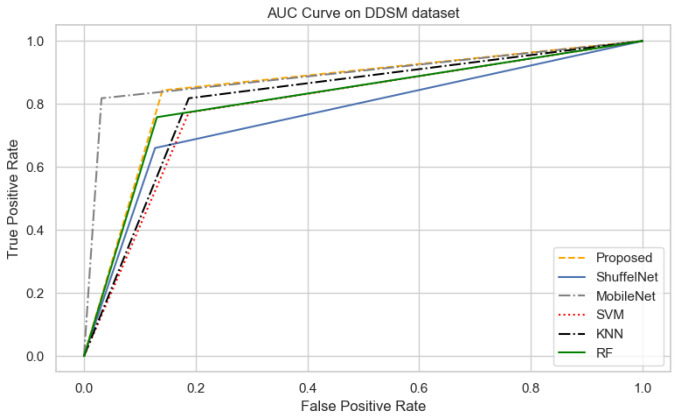
The AUC curves of algorithms on DDSM Dataset.

On the other hand, when comparing the sensitivity of the proposed model with ShuffelNet, MobileNet, SVM, K-NN, and RF on the PINUM and CBIS-DDSM is 3% 13%, 11%, 8%, 2%, 1%, 1%, and 16%, 13%, 1% higher, respectively as shown in Figure 19 and Figure 20. Figure 21 reveals that the sensitivity of the proposed method on the DDSM dataset was 7%, 8%, 15%, 15%, and 6% higher than ShuffelNet, MobileNet, SVM, K-NN, and RF. The area under the curve (AUC) was calculated of our proposed model, as shown in Figure 22, Figure 23 and Figure 24. The AUC curve of our model was higher than the ShuffelNet, MobileNet, SVM, K-NN, and random forest. The above aforementioned deep analysis of all datasets stated that the proposed method significantly outperforms rather than the ShuffelNet, MobileNet, SVM, K-NN, and RF. The experimental results demonstrated the effectiveness of a deep convolutional neural network to classify architectural distortion ROIs that can help doctors and radiologists to predict breast cancer at initial stages.

### 5.3. Results Comparison between Proposed Method and Previous Studies

The proposed method is validated by comparing it with previous studies using the same dataset and the private dataset. The experimental results reveal that the performance of the proposed method was much better than the previous studies. Table 8 summarized that the proposed method was achieved 0.95, 0.97, and 0.98 accuracies on the PINUM, CBIS-DDSM, and DDSM datasets, respectively, which were higher comparatively from previous studies. Murali. et al. [6] pertain SVM and MLP for classifying architectural distortion ROIs and achieved 89.6% accuracy on the DDSM dataset. [7] implemented the Gober filter-based method to detect architectural distortion and achieve 90% sensitivity. The authors [8,9,10] employed a machine learning-based classification algorithm to detect architectural distortion from the DDSM data set and reporting 83.50%, 92.94%, and 91.79% accuracies, respectively. Another study by [13] applied a multilayer-perception network to detect architectural distortion evaluating 300 images and reported 83% accuracy. The authors [14] used the LDA classifier to detect architectural distortion tracking from digital breast tomosynthesis and achieved 0.90 sensitivity.

The proposed method depth-wise 2D convolutional neural network achieved 0.95, 0.97, and 0.98 accuracies on the 3264 PINUM, 3568 CBIS-DDSM, and 5500 DDSM datasets images, respectively, which were better than previous studies. The proposed model has achieved 0.98 accuracy which was 6% and 15% higher than the previous studies on the DDSM dataset which indicates that the performance of the proposed modal was much better. The performance of the proposed method on a private dataset was also better than the previous studies.

## 6. Discussion

In the current study, proposed a state-of-the-art computer-aided diagnostic system using a computer vision and depth-wise 2D convolutional neural network to detect and classify architectural distortion ROIs from digital mammograms. The proposed mammogram classification framework pertains to four steps: image preprocessing and augmentation, image pixel-wise segmentation, architectural distortion ROI’s detection, training deep learning, and machine learning networks to classify AD’s ROIs into malignant and benign classes. Image classification using the deep convolutional neural network, a minimum number of images is approximately 1000 required, and it can be increased for pre-trained models to regularize the neural network. [56]. Deep learning is a data-driven method so that the small size of data and non-standardization are the main challenges for the generalization of the model. However, to handle the generalization, overfitting, and improving the robustness of the deep learning model, we artificially inflate the PINUM database up to 3462 using data augmentation techniques as discussed above. The CBIS-DDSM dataset consists of 3568 mammogram images, including 1740 benign and 1828 malignant images with MLO and CC views. The 5500 images were included from the DDSM dataset. Split the data into the training, testing, and validation data for the proposed deep neural modal. The dataset was randomly divided into 60% for training, 20% for testing, and 20% for cross-validation.

In the context of comparing results with the ShuffleNet, MobileNet, SVM, K-NN, and RF the obtained results of the proposed method are comparable, encouraging, and better in many aspects. The proposed method yielded better accuracy, f1-score, precision, recall, sensitivity, and area under the curve. When we are seeing the training accuracy of the proposed method on both datasets it reaches 100% as compared with the ShuffelNet and MobileNet. On the other hand, the training loss of our proposed method is consistently decreasing after the 7th epochs which shows the noise around the proposed method is much lower than the ShuffleNet and MobileNet on the PINUM, CBIS-DDSM, and DDSM datasets. In comparison to the findings of previous research on architectural distortion, the current study’s findings for malignant and benign ADs are promising, better, and outperforms. The authors [6,8,9,10] achieved 89.6%, 83.50%, 92.94%, and 91.79% accuracies, respectively. The experimental results demonstrated that the proposed approach significantly outperforms the ShuffelNet, MobileNet, SVM, K-NN, RF, and previous studies. The proposed approach achieved 0.95%, 0.97%, 0.98% accuracies on the PINUM, CBIS-DDSM, and DDSM dataset, while the maximum accuracy in previous studies was 92.94% [9] on the DDSM dataset, which healed our model. On the other hand, the highest accuracy was achieved by the random forest algorithm are 0.90, 0.95 on the PINUM and CBIS-DDSAM dataset, which is still lower than our proposed model. Furthermore, to enhance the effectiveness of the proposed model, compared it with other evaluation metrics such as f1-score, precision, recall, and sensitivity; the model achieved better results, as seen in Table 5, Table 6 and Table 7.

Fully automatic identification of architectural distortion in mammograms of interval-cancer cases is more challenging because extensive comparative analysis, which was not investigated in our study, is still a limitation. The diagnostic mammograms were not accessible in the current investigation on interval-cancer patients, including benign control cases, because of localizing the areas of architectural distortion on mammograms.

The current study observed that the classification approach using depth-wise 2D convolutional neural networks was much better than the machine learning algorithms such as SguffelNet, MobileNet, SVM, K-NN, and RF. Moreover, computer-vision technology is more potent for image segmentation and ROIs detection than the traditional and hand-crafted approaches. The proposed fully automated CAD system could predict breast cancer more accurately than the older one and help the clinical staff with disease diagnostic. To enhance the validity of the model, employed it on the three databases, the public and the private. The proposed approach with a computer vision and depth-wise 2D convolutional neural network is a novel approach for architectural distortion ROIs detection and classification into benign and malignant ROIs.

## 7. Conclusions

Mammogram screening is an effective and initial screening method for the diagnosis of breast cancer in women. Architectural distortion is the third most suspicious appearance on a mammogram that represents abnormal regions. Architectural distortion detection from mammograms is challenging due to its subtle and varying asymmetry on breast mass and small size. Therefore, the manual interpretation of Architectural Distortion is a challenging task for radiologists to figure out abnormalities during the examination of mammograms due to its subtle appearance on fatty denser mass. In the current study, proposed an automated computer-aided diagnostic system based on computer vision and deep learning to predict breast cancer from the digital mammogram. Proposed a state-of-the-art- method for breast cancer detection from architectural distortion ROIs. The proposed method consists of two major phases, in the first phases the architectural distortion ROIs are extracted using a computer vision algorithm and verified by the expert radiologists, in the 2nd phase these ROIs are classified with the proposed deep learning method to classify into malignant and benign ROIs. Experimental results reveal that our proposed method outperforms as compared with the ShuffelNet, MobileNet, SVM, K-NN, RF, and previous studies. Although the results are very promising and better, further investigate new techniques for localizing the patterns for detecting architectural distortion ROIs that are not limited to spiculated patterns. Furthermore, will investigate other deep learning models to detect architectural distortion from other public and larger private datasets. In addition, we will also analyze our modal to improve the true-positive rate and detect ADs tracks from DBT slices. Another, limitation to this study is the use of transfer learning for handling small label datasets which will be further considered in future studies. 

## Figures and Tables

**Table 1:** Data Set Description and Detail.

Mammogram Label	Category	Images	Dataset
Benign (0)	Original	425	PINUM
Malignant (1)	Original	152	PINUM
Benign (0)	Augmented	2550	PINUM
Malignant (1)	Augmented	912	PINUM
Benign (0)	AD ROIs	75	PINUM
Malignant (1)	AD ROIs	75	PINUM
Benign (0)	Original	1740	CBIS-DDSM
Malignant (1)	Original	1828	CBIS-DDSM
Benign (0)	AD ROIs	100	CBIS-DDSM
Malignant (1)	AD ROIs	100	CBIS-DDSM
Benign (0)	Original	2500	DDSM
Malignant (1)	Original	3000	DDSM
Benign (0)	AD ROIs	100	DDSM
Malignant (1)	AD ROIs	100	DDSM

**Table 2:** Data augmentation techniques with performance value.

Sr	Augmentation Techniques	Performance Values
1	Rotation	45${}^{\circ }$, 90${}^{\circ }$, 135${}^{\circ }$, 180${}^{\circ }$, 360${}^{\circ }$
2	Flipping	Left, Right, Top, Bottom
3	Sharpen (lightness value)	0.5–1.5
4	D-skew (angle)	15${}^{\circ }$, 40${}^{\circ }$
5	Contrast (intensity value)	20–60%
6	Brightness (darkness values)	15–55%

**Table 3:** The proposed network layers architecture.

Network Layers	Filters	Filter Size	Padding	Stride	Output Shape
Input Image	-	-	-	-	240×320×3
DW_Conv2D	64	3×3×64	same	1×1	100×100×64
Activataion_Relu	-	-	-	-	98×98×64
Max_Pooling	1	2×2	-	0	49×49×64
DW_Conv2D	64	3×3×64	same	1×1	47×47×64
Activataion_Relu	-	-	-	-	47×47×64
Max_Pooling	1	2×2	-	0	23×23×64
DW_Conv2D	64	3×3×64	same	1×1	21×21×64
Activataion_Relu	-	-	-	-	21×21×64
Max_Pooling	1	2×2	-	0	10×10×64
Dropout (0.5)	-	-	-	-	10×10×64
FC1_Flatten_4	-	-	-	-	(6400)
FC2_Dense_5	64	-	-	-	(6400)
Sigmoid	-	-	-	-	[0/1]

**Table 4:** Hyper parameter configuration detail.

Configuration	Values
Batch Size	16
Learning Rate	0.001
Epochs	20
Optimization function	Adam
Loss Function	binary_crossentropy
Target Size	[320, 240]
histogram_freq	1
Tarin Split	0.6
Validation Split	0.2

**Table 5:** Performance Evaluation compression of proposed method and with ShuffelNet, MobileNet, SVM, K-NN and RF on PINUM dataset.

Algorithms	Accuracy	F1-Score	Precision	Recall	Sensitivity	AUC
Proposed	0.95	0.87	0.90	0.89	0.99	0.91
ShuffelNet	0.91	0.81	0.86	0.76	0.95	0.79
MobileNet	0.89	0.77	0.61	0.73	0.85	0.79
SVM	0.87	0.72	0.88	0.61	0.97	0.69
KNN	0.83	0.63	0.84	0.51	0.96	0.59
RF	0.90	0.81	0.89	0.75	0.96	0.75

**Table 6:** Performance Evaluation compression of proposed method and with ShuffelNet, MobileNet, SVM, K-NN and RF on CBIS-DDSM dataset.

Algorithms	Accuracy	F1-Score	Precision	Recall	Sensitivity	AUC
Proposed	0.97	0.96	0.94	0.98	0.95	0.98
ShuffelNet	0.93	0.69	0.75	0.73	0.84	0.69
MobileNet	0.90	0.93	0.82	0.83	0.87	0.61
SVM	0.73	0.69	0.73	0.66	0.79	0.67
KNN	0.80	0.78	0.79	0.78	0.82	0.81
RF	0.95	0.95	0.93	0.97	0.94	0.89

**Table 7:** Performance Evaluation compression of proposed method and with ShuffelNet, MobileNet, SVM, K-NN and RF on DDSM dataset.

Algorithms	Accuracy	F1-Score	Precision	Recall	Sensitivity	AUC
Proposed	0.98	0.90	0.96	0.86	0.96	0.85
ShuffelNet	0.87	0.74	0.83	0.76	0.89	0.69
MobileNet	0.90	0.84	0.85	0.84	0.88	0.81
SVM	0.80	0.76	0.74	0.77	0.81	0.79
KNN	0.81	0.78	0.75	0.82	0.81	0.81
RF	0.91	0.88	0.95	0.82	0.90	0.78

**Table 8:** Comparison of results with previous studies and proposed method.

Authors	Problem	Method	Database	Images	Accuracy
[6]	Architectural Distortion Detection	SVM, MLP	DDSM	190	0.89
[7]	Architectural Distortion Detection	Bayesian, SELF ANN	Private	1745	N/A
[8]	Architectural Distortion Detection	Differential direction method	DDSM	33	0.83
[9]	Architectural Distortion Detection	SVM	DDSM	147	0.92
[10]	Architectural Distortion Detection	Sparse classifier	DDSM	69	0.91
[13]	Architectural Distortion Detection	MLP	FFDM	300	0.83
[14]	Architectural Distortion tracking	LDA	FFDM	37	N/A
[55]	Architectural Distortion tracking	CNN	CBIS-DDSM	334	0.92
Proposed	Architectural Distortion Detection	Depth-wise 2DCNN	Private (PINUM)	3462	0.95
Proposed	Architectural Distortion Detection	Depth-wise 2DCNN	CBIS-DDSM	3568	0.97
Proposed	Architectural Distortion Detection	Depth-wise 2DCNN	DDSM	5500	0.98

## Data Availability

The CBIS-DDSM [49] and DDSM [50] dataset is publicly available and the Private PINUM [48] data set is collected from local hospital.
